# Effect of expansion of human umbilical cord blood CD34 + cells on neurotrophic and angiogenic factor expression and function

**DOI:** 10.1007/s00441-022-03592-2

**Published:** 2022-02-02

**Authors:** Ashalyn P. Watt, Mark Kirkland, Lakshmi Nekkanti, Yen Pham, Courtney McDonald, Atul Malhotra, Guy Moeneclaey, Suzanne L. Miller, Graham Jenkin

**Affiliations:** 1 The Ritchie Centre, Hudson Institute of Medical Research, Clayton, VIC Australia; 2 Cell Care Australia, Heatherton, VIC Australia; 3 Institute for Frontier Materials, Deakin University, Waurn Ponds, Victoria, Australia; 4 Department of Paediatrics, Monash University, Clayton, VIC Australia; 5 Department of Obstetrics and Gynaecology, Monash University, Clayton, VIC Australia

**Keywords:** CD34, Haematopoietic stem cells, Cell expansion, Neurotrophins, Angiogenesis

## Abstract

The use of CD34 + cell-based therapies has largely been focused on haematological conditions. However, there is increasing evidence that umbilical cord blood (UCB) CD34 + -derived cells have neuroregenerative properties. Due to low cell numbers of CD34 + cells present in UCB, expansion is required to produce sufficient cells for therapeutic purposes, especially in adults or when frequent applications are required. However, it is not known whether expansion of CD34 + cells has an impact on their function and neuroregenerative capacity. We addressed this knowledge gap in this study, via expansion of UCB-derived CD34 + cells using combinations of LDL, UM171 and SR-1 to yield large numbers of cells and then tested their functionality. CD34 + cells expanded for 14 days in media containing UM171 and SR-1 resulted in over 1000-fold expansion. The expanded cells showed an up-regulation of the neurotrophic factor genes BDNF, GDNF, NTF-3 and NTF-4, as well as the angiogenic factors VEGF and ANG. In vitro functionality testing showed that these expanded cells promoted angiogenesis and, in brain glial cells, promoted cell proliferation and reduced production of reactive oxygen species (ROS) during oxidative stress. Collectively, this study showed that our 14-day expansion protocol provided a robust expansion that could produce enough cells for therapeutic purposes. These expanded cells, when tested in in vitro, maintained functionality as demonstrated through promotion of cell proliferation, attenuation of ROS production caused by oxidative stress and promotion of angiogenesis.

## Introduction

Umbilical cord blood (UCB) contains a mixed population of stem and progenitor cells that includes haematopoietic stem cells (HSCs), endothelial progenitor cells (EPCs) and mesenchymal stromal cells (MSCs) (Phuc et al. 2012; Roura et al. 2015), together with immunosuppressive cells such as myeloid-derived suppressor cells (MDSC) and regulatory T cells (Tregs) (Tolar et al. 2009; Gervassi et al. 2014). This range of cell populations possess a diverse mix of multi-functional capacities to mediate tissue regeneration and repair, and combined with the straightforward nature of cord blood collection, makes UCB-derived cells a strong option for cell-based therapies. HSCs are commonly identified by their expression of the cell surface protein CD34, and HSCs make up between 0.5 to 1% of the total mononuclear cells (MNCs) population in cord blood collected at term birth (Kinniburgh and Russell 1993; Sutherland et al. 1994; Van Epps et al. 1994; Krause et al. 1996). UCB-HSCs are more primitive and have a lower risk of rejection compared to HSCs obtained from bone marrow, even when considering allogeneic use. This is because UCB-HSCs have low levels of HLA antigens which are critical in the mediation of graft-versus-host disease (Rocha et al. 2000; Tse et al. 2008; Park and Won 2009). Importantly, the concentration of CD34 + cells is tenfold greater in human cord blood when compared to peripheral blood (Murohara et al. 2000). In light of this, it is not surprising that UCB transplants are now being utilised in clinical settings for the treatment of a wide range of haematological and non-haematological diseases (Gluckman et al. 1989; Bradstock et al. 2006; Hügle and Daikeler 2010; Sebrango et al. 2010; Thompson et al. 2012; Shenoy 2013; Swart et al. 2017).

UCB-derived MNCs and UCB-derived HSCs are currently under clinical investigation for a wide range of regenerative medicine applications, with > 70 clinical trials currently registered globally (Clinicaltrials.gov). Many of these regenerative or reparative trials are based around the therapeutic application for MNCs or HSCs to mediate neuropathology, across a spectrum of adult, childhood and neonatal conditions. These trials are predominantly centred around the capability of UCB-derived cells, including CD34 + haematopoietic stem cells and their progeny, to upregulate neurotrophic factors. A study by Paczkowska et al. assessed cord blood CD34 + cells for expression of neurotrophic factors and showed that these CD34 + cells express brain-derived neurotrophic factor (BDNF), neurotrophin-3 (NTF-3), neurotrophin-4 (NTF-4), nerve growth factor (NGF) and glial-derived neurotrophic factor (GDNF). These cells also express the neurotrophic receptors; neurotrophic receptor tyrosine kinase 1 (TrkA), neurotrophic receptor tyrosine kinase 2 (TrkB), neurotrophic receptor tyrosine kinase 3 (TrkC) and nerve growth factor receptor (p75NTR) (Paczkowska et al. 2013). Aside from their engraftment potential and an established ability to restore the immunological system, HSCs secrete a range of proteins that regulate apoptosis and promote proliferation and repair, including cytokines, chemokines, anti-inflammatory factors and vascular growth factors (Aguila et al. 1997; Majka et al. 2001; Shizuru et al. 2005; Baraniak and McDevitt 2010; Czechowicz and Weissman 2011).

The majority of studies undertaken to characterise the neuroprotective or neuroreparative potential of HSCs have been performed in adult models of stroke and/or ischemia (Taguchi et al. 2004; Boltze et al. 2005; Shyu et al. 2006; Schäbitz et al. 2007; Schwarting et al. 2008; Felfly et al. 2010). The neuro-modulatory mechanisms of action of HSCs are also of interest as a treatment for neonatal brain injury, given that neonatal encephalopathy is often mediated via hypoxic ischemic injury that might benefit from the potential angiogenic and neurogenic properties exhibited by HSCs. In studies undertaken by our group, we have demonstrated that UCB provides neuroprotection in a neonatal hypoxic ischemic model (McDonald et al. 2018). In other studies, the administration of cord blood-derived CD34 + cells following neonatal hypoxic ischemic brain injury in mice and rats demonstrates that cell treatment may be protective, noting improved cerebral blood flow, a reduction in apoptosis, vascular regeneration and improved motor function (Ohshima et al. 2016; Yu et al. 2019). These data collectively suggest that HSCs have excellent neuroprotective potential for the immature brain; however, this is conditional on sufficient numbers of functional CD34 + cells being obtained.

Although umbilical cord blood is a rich source of HSCs compared to bone marrow and peripheral blood, the number of HSCs obtained from one cord blood sample at term birth may not be sufficient for therapeutic use in regenerative medicine applications, especially in adults and/or where multiple administrations are indicated (Qiu et al. 2021). In preclinical studies of neonatal brain injury, multiple doses of UCB-MNCs are more effective than a single dose of cells in reducing long-term neuroinflammation, cerebral tissue damage, and in improving behavioural outcomes (Penny et al. 2020). This highlights the need to develop methodologies to produce large numbers of functional UCB-derived cells, thus requiring a robust cell expansion protocol. Accordingly, in the current study we set out to optimise an expansion technique for CD34 + HSCs derived from pooled UCB CD34 + HSCs, to maximise the number of functional CD34 + cells obtainable. There has been considerable effort placed in identifying factors for use in the ex vivo expansion of CD34 + cells to promote proliferation and self-renewal. This includes the use of growth factors, inhibitors and small molecules (as reviewed by (Flores-Guzmán et al. 2013; Xie and Zhang 2015; Zhang and Gao 2016)); the addition of cytokines including, interleukin 6 (IL-6), stem cell factor (SCF), thrombopoietin (TPO), interleukin 3 (IL-3) and FMS-like tyrosine kinase 3 (FLT-3) and to the culture media (Petzer et al. 1996); and modulating oxygen levels under culture conditions (Tiwari et al. 2018). More recently, attention has turned to the use of small molecules to modulate stem cell fate, with StemRegenin-1 (SR-1) inclusion to TPO, IL-6, FLT-3 and SCF-containing media shown to induce a 50-fold increase in the number of HSCs obtained (Boitano et al. 2010). Furthermore, UM171 added to SCF, FLT-3, TPO and low-density lipoprotein (LDL) containing media has been shown to enable expansion of HSCs by up to 100-fold over 7 days of culture (Fares et al. 2014). Thus, the addition of these small molecules has led to progress in the clinical application of small molecule expanded HSCs for haematological uses (Wagner et al. 2016; Cohen et al. 2020; Dumont-Lagacé et al. 2020). It is, however, unknown if the addition of small molecules used during ex vivo expansion affects the production and expression of angiogenic and neurotrophic factors which, in turn, could impact the efficacy of expanded HSCs in neuroregenerative medicine. We hypothesised that UCB-derived CD34 + cell expansion using our protocol would not affect neurotrophic and angiogenic factor gene expression and that expanded CD34 + HSCs would retain their functional ability to promote proliferation and their neurotrophic and angiogenic activity.

## Materials and Methods

### Ethics

We obtained human ethics approval from Monash Health Human Ethics Committee, Monash Medical Centre for umbilical cord blood collection from term pregnancies (HREC Ref. Research Project No.: 12387B). The experiments that involved use of animals had ethics approval from Monash Medical Centre Animal Ethics Committee A (MMCA/2015/42). All experiments followed Australian National Health and Medical Research Council guidelines.

### Umbilical cord blood collection and isolation of cord blood CD34 + cells

Human umbilical cord blood samples were obtained from women with uncomplicated pregnancies giving birth at term via elective caesarean section (> 37 weeks gestation), with all women providing written informed consent. After clamping of the cord and delivery of the placenta, UCB was collected from the umbilical vein using blood collection bags that contained anticoagulant (Macopharma). We obtained an average of approximately 80 ml of UCB. To obtain the mononuclear fraction of cells, UCB was diluted 1:1 with PBS/2 mM EDTA and layered onto 50-ml Sepmate tubes (Stem Cell Technologies) that contained 13 ml Ficoll-Paque Plus (GE Healthcare) and this was centrifuged at 1200* g* for 15 min. MNCs were washed twice with PBS/2 mM EDTA and centrifuged at 200* g* for 10 min to produce a cell pellet that was collected in a 50-ml centrifuge tube. We used the trypan blue exclusion dye (Gibco) to assess cell viability, and cell number was quantified using a haemocytometer. The MNCs were then used for positive CD34 selection using a MACS bead separation system (Miltenyi Biotec), with all procedures performed according to manufacturer’s instructions. Following isolation, CD34 + purity was quantified via flow cytometry and the isolates used were confirmed to have greater than 90% purity. Isolated UCB CD34 + cells were cryopreserved at a density of 1 × 10^6^ cells/ml in recovery cell culture freezing medium (Gibco) and stored in a vapour phase liquid nitrogen until required.

### Expansion of CD34 + cells

CD34 + cells from up to 10 umbilical cord blood samples were thawed and pooled for expansion studies. The pooled CD34 cells were cultured in serum-free Stemspan Animal Component-Free (ACF) media (Stem Cell Technologies) supplemented with 1% antibiotic/antimycotic (Life Technologies) and standard growth factors (SGF) consisting of 50 ng/ml SCF, 50 ng/ml TPO, 80 ng/ml FLT-3 and 100 ng/ml IL-6 (Miltenyi Biotec). Combinations of the following were added to the SGF media; 20 µg/ml LDL, 750 nM SR-1 (Boitano et al. 2010) and UM171 (Stem Cell Technologies) at doses ranging from 35 to 400 nM. CD34 + cells were seeded in triplicate in 24-well tissue culture-treated plates (Corning) at 1 × 10^4^ cells/well/1 ml and cultured in a dynamic setting by placing the plates on an orbital shaker at 20 rpm in a 37℃ incubator with 5% CO_2_ and 5% O_2._ The cells were cultured over 7 days with a media top-up on day 5, before either being harvested at day 7 for analysis or split at a ratio of 1:20 and cultured for a further 7 days in the above media.

### Colony-forming unit (CFU) assays

Colony-forming unit (CFU) assay was carried out for pre- and post-expanded cells using complete Methocult media (Stem Cell Technologies) following the manufacturer’s instructions. The cells were resuspended in Methocult and seeded in triplicate onto 35 mm non-tissue culture-treated dishes at 1,000 cells/mL and cultured at 37℃ with 5% CO2 for 14 days. The colonies were then manually counted using a light microscope (Olympus) to identify the burst-forming unit erythroid (BFU-E), colony-forming unit granulocyte, macrophage (CFU-GM), colony-forming unit granulocyte (CFU-G), colony-forming unit macrophage (CFU-M) and colony-forming unit granulocyte, erythrocyte, macrophage, megakaryocyte (CFU-GEMM) cells.

### Flow cytometry

#### Phenotypic analysis

After 7 and 14 days of culture, the expanded cells were analysed for CD34 purity. Flow cytometric analysis for surface markers was performed using the following antibodies: CD34 PE, CD45 APC, 7AAD, CD133 BV421, CD43 BV510 and CD90 PE-CY7 (all antibodies from BD Biosciences). Single stains were used for compensation and fluorescence minus one (FMO) was used for setting gates. Antibody-stained cells were assessed using a FACS Canto II (BD Biosciences) flow cytometer, using FACSDiva software (BD Biosciences). Mean and standard error values were determined from at least three independent experiments, with each experiment performed in triplicate. Fold expansion of CD34 + and other lineage-specific cells after 7 and 14 days of culture was calculated by comparing the proportion of cells, compared to the initial seeding of 1 × 10^4^ cells.

#### Cell cycle analysis

Cell cycle analysis was conducted using 7AAD staining. Cells were first fixed by adding ice cold 80% ethanol via a dropper and then incubated for 2 h at -20 ℃. Fixed cells were washed three times with FACS buffer (2% FBS in 1 × PBS) treated with RNase and stained with 7AAD for 30 min. Cells were analysed in an FACS Canto II (BD Biosciences) flow cytometer using FACSDiva software.

### Tube formation assay

Human umbilical vein endothelial cells (HUVECs) (Life Technologies) were cultured in medium 200 with 1 × LVES supplement (Life Technologies). For the tube formation assay, 200 µl of thawed geltrex (growth factor reduced) was added to the bottom of a 24 well plate and allowed to set for 1 h in a 37 °C incubator. HUVECs (2 × 10^4^) were plated in medium 200 basal media with no supplements and 2 × 10^5^ CD34 + cells placed in a transwell (0.4 µM pore) above the culture and incubated for 24 h. Images were taken on an Olympus CX53 microscope analysed using the Image J angiogenesis tool.

### Glial cell proliferation assay

A population of mixed glial cells were isolated from the cortex of 3-day old rat pup brains. At post-mortem, brains were dissected and cortical tissue separated and digested using a neural tissue dissociation kit (P) (Miltenyi Biotec), following manufacturer’s specifications. Single cells were plated on poly-l-ornithine coated flasks in growth media containing DMEM, 2 × B27 supplement and 1 × antibiotic/antimycotic in a 37℃ incubator with 5% CO_2_. After one week in culture, the cells were dissociated with accutase solution (Life Technologies) and plated in 24 well plates pre-coated with poly-l-ornithine at a density of 5 × 10^3^ cells per well in media containing DMEM and 1 × B27 supplement. Expanded CD34 + cells were placed above the cells in a transwell polycarbonate insert with a 0.4 µM pore at a density of 1 × 10^5^ cells. Proliferation of the glial cells was measured using the CyQuant cell proliferation assay (Life Technologies) following the manufacturer’s specifications.

### Oxidative stress and ROS production

To assess ROS production induced by oxidative stress in the mixed glial cells, a DCFDA kit (Abcam) was used following manufacturer’s specifications. Glial cells were stained for 30 min with 20 µM DCFDA, and then tert-butyl hydroperoxide (TBHP) was added at a concentration of 5 µM to induce oxidative stress (Roy and Sil 2012) with/without the addition of pre- or post-expanded CD34 + cells placed in a transwell for 24 h. Fluorescence was measured using a microplate reader with an ex/em 485/535 nm.

### Microglia anti-inflammatory assay

Microglia were isolated from P3 rat pup brain cortices using MACS bead separation for positive selection of CD11b (Miltenyi Biotec). Once isolated, microglia were cultured in poly-D-lysine coated tissue culture flasks in serum free conditions of 50% DMEM/50% Neurobasal media, 1% N2 supplement, 1% antibiotic/antimycotic (all from Life Technologies). Once the cells reached 80% confluence, they were plated in 24 well plates (4 × 10^4^ cells/well/0.5 ml) and challenged with 100 ng/ml lipopolysaccharide (LPS) from Escherichia coli O111:B4 (Sigma Aldrich) with/without the addition of 2 × 10^5^ CD34 + cells, placed in a transwell (0.4 µM pore) above the culture. After 24 h, RNA was extracted from the microglial cells for gene expression analysis.

### RNA extraction and cDNA synthesis

RNA was extracted from pooled CD34 + cells pre- and post-expansion (n = 9 pooled donors) using a PureLink RNA extraction kit following the manufacturer’s instructions, including the optional on-column DNase treatment using PureLink DNase set (Life Technologies). cDNA was synthesised from RNA extracted from CD34 + cells following the iScript reverse transcriptase protocol (Bio-Rad). RNA and cDNA concentrations were measured using a nanodrop spectrophotometer (Thermo Fisher) and stored at -80 ℃ or -20 °C, respectively, until required.

### Gene expression analysis

qPCR was conducted on cDNA from pre- and post-expanded CD34 + cells and was used to identify the expression of neurotrophic and angiogenic-related genes. Gene specific primers.

were designed (Table 1) using glyceraldehyde-3-phosphate dehydrogenase (GAPDH) and/or beta-2 macroglobulin (B2M) as the housekeeping gene for normalisation. PCRs were carried out using the Quant studio 6 Flex Real-Time PCR system (Applied Biosystems). Ten microlitres of reaction mixtures contain 5 µl SYBR Green PCR master mix (Applied Biosystems), 50 ng cDNA and 250 nM forward and reverse primers. The relative gene expression was determined using the comparative Ct method.

**Table 1:** Primer list

Primer name	Sequence	Product size
Hu-GAPDH-fwd	AGAAGGCTGGGGCTCATTTG	82
Hu-GAPDH-rev	ATGACGAACATGGGGGCATC	
Hu-B2M-fwd	GGGTTTCATCCATCCGACA	218
Hu-B2M-rev	GCTTACATGTCTCGATCCCAC	
Hu-ANGPT1-fwd	TGGGGGAGGTTGGACTGTAA	149
Hu-ANGPT1-rev	GCCTCTGACTGGTAATGGCA	
Hu-ANG-fwd	TTCTGCGGACTTGTTCTGAG	148
Hu-ANG-rev	GTCAGGAAGTGTGTGTACCTG	
Hu-NTF3-fwd	GGTACGCGGAGCATAAGAGTC	141
Hu-NTF3-rev	GAGAGTTGCCCGTTTTGATCT	
Hu-NTF4-fwd	GCGAGGTGGAGGTGTTGG	100
Hu-NTF4-rev	CCTTCCTCAGCGTTATCAGC	
Hu-VEGFA-fwd	TGCAGATTATGCGGATCAAACC	81
Hu-VEGFA-rev	TGCATTCACATTTGTTGTGCTGTAG	
Hu-GDNF-fwd	TGACTTGGGTCTGGGCTATG	174
Hu-GDNF-rev	TCATCAAAGGCGATGGGTCT	
Hu-BDNF-fwd	GATGCTCAGTAGTCAAGTGCC	168
Hu-BDNF-rev	GCCGTTACCCACTCACTAATAC	

### Statistics

Statistical analysis was carried out using GraphPad Prism software, and all data are presented as mean ± standard error of the mean. Analysis of CD34 + expansion, glial cell proliferation, tube formation assays and gene expression analysis were conducted using a one-way ANOVA and where appropriate a two-way ANOVA, both with a Tukey post hoc test. Significance was annotated as P < 0.05 *, P < 0.01 ** and P < 0.001 *** or no significance ns.

## Results

### CD34 + 7 versus 14 day expansion

Firstly, we compared our standard 7-day expansion versus a 14-day expansion with a 1:20 split at day 7, using media containing standard growth factors (SGF) (TPO, SCF, IL-6 and FLT-3) or in addition, a combination of the following molecules: UM171 (35 nM), SR-1 (750 nM) and LDL (20 µg/ml). At 7 days of expansion, the CD34 + cells were assessed for CD34/CD45 expression (Table 2). After 7 days of expansion, the highest fold expansion of CD34 + /CD45 + cells was observed in the group containing both UM171 and LDL (45.1 ± 1.6); however, the percentage of cells expressing CD34/CD45 was highest in the group containing UM171 and SR-1 (68.1 ± 2.7). This indicates that the combination containing LDL promoted more proliferation but caused further maturation of the cells.

**Table 2:** Assessment of 7- and 14-day CD34 + expansion

Condition	Time of culture (days)	Total cell counts (× 10^6^)	CD34 + CD45 + cell positivity (%)	Fold expansion CD34 + CD45 +
**SGF**	7	0.625 ± 0.048	27.9 ± 3.3	17.6 ± 2.3
	14	6.958 ± 0.57*	20.0 ± 1.9*	99.3 ± 11.9*
**UM171**	7	0.54 ± 0.022	56.7 ± 3.0	32.0 ± 1.7
	14	8.568 ± 0.73*	35.2 ± 1.3*	300.2 ± 36.3*
**SR-1**	7	0.644 ± 0.021	44.1 ± 2.5	28.8 ± 1.5
	14	8.627 ± 0.92*	31.8 ± 0.7*	244.2 ± 31.3*
**LDL**	7	0.699 ± 0.020	32.7 ± 2.8	22.1 ± 1.7
	14	9.984 ± 0.85*	23.8 ± 1.1*	170.7 ± 14.5 *
**UM171 + SR-1**	7	0.551 ± 0.02	68.1 ± 2.7	38.9 ± 0.96
	14	12.28 ± 1.11*#	42.3 ± 2.7*#	532.0 ± 73.3*#
**UM171 + LDL**	7	0.754 ± 0.023	57.6 ± 2.6	45.1 ± 1.6
	14	11.46 ± 0.78*	35.5 ± 1.8*	392.8 ± 47.7*
**SR-1 + LDL**	7	0.801 ± 0.048	42.5 ± 4.0	32.6 ± 1.8
	14	11.558 ± 1.02*	30.6 ± 1.3*	289.1 ± 37.3*

*P < 0.05 compared to day 7

**P < 0.05 UM171 + SR1 compared to all other groups

At day 7, the cells were split at a 1:20 ratio and cultured for a further 7 days to increase cell yield. At 14 days, the cell cultures were assessed for total cell counts, CD34 + /CD45 + positivity, and total CD34 + /CD45 + fold expansion. After 14 days of culture, all groups had significantly higher total cell counts and CD34 + /CD45 + fold expansion compared to day 7; however, all groups had significantly lower CD34 + /CD45 + positivity in the cultures compared to pre-expansion and day 7. After 14 days of expansion, the group containing UM171 and SR-1 resulted in the highest number of cells in the cell counts, as well as fold expansion (532.0 ± 73.3) and CD34 + /CD45 + expression (42.3 ± 2.7) compared to all other groups. As the addition of UM171 + SR-1 resulted in the highest expansion rate, all further experiments were conducted using this combination.

### Effect of UM171 dose on CD34 + expansion

To further assess the effect of UM171 concentration on CD34 + expansion, UM171 was added to SGF at the following concentrations: 50 nM, 100 nM, 150 nM, 200 nM and 400 nM, along with SR-1 at 750 nM (Table 3). At day 7, the highest fold expansion of CD34 + /CD45 + cells was observed in the group containing 150 nM UM171 (75.3 ± 5.8). However, the highest CD34 + /CD45 + positivity was observed in the group containing 400 nM UM171 (93.0% ± 0.9), but this concentration also had a significantly lower fold expansion than all other groups (49.6 ± 0.4). By day 14 of expansion, all groups had significantly higher cell counts and an increase in fold expansion of CD34 + /CD45 + cells when compared to day 7 of expansion. Two groups resulted in over 1000 fold expansion of CD34/CD45 + cells after 14 days, these were UM171 at 100 nM (1167.3 ± 89.6) and 150 nM (1120.5 ± 90.6). The highest CD34 + /CD45 + cell purity post-expansion at 14 days was observed when UM171 was used at 400 nM (87.7 ± 0.7); however, at this dose there was a significant impact on cell proliferation, resulting in lower cell counts and a lower CD34 + /CD45 + fold expansion (643.8 ± 5.6). These results support that CD34 + /CD45 + positivity after expansion increased with increasing UM171 concentrations. In contrast, fold expansion significantly declined as UM171 concentration increased at 200 nM and above. Taken collectively, although UM171 at 100 nM provided the highest fold expansion of CD34 + /CD45 + cells, there was no significant difference between fold expansion of CD34 + /CD45 + cells between UM171 at 100 nM and 150 nM (P = 0.724). However, UM171 at the concentration of 150 nM resulted in significantly higher CD34 + /CD45 + positivity compared to UM171 at 100 nM (78.9% and 71.5%, respectively, P = 0.039), so the concentration of UM171 at 150 nM was used for all further experiments.

**Table 3:** Effect of UM171 dose on CD34 + expansion at 7 and 14 days

UM171 concentration (nM)	Time of culture (days)	Total cell counts (× 10^6^)	CD34 + CD45 + cell positivity (%)	Fold expansion CD34 + CD45 +
**50**	7	0.802 ± 0.024	77.8 ± 0.8	68.3 ± 12.0
	14	13.28 ± 0.357*	52.5 ± 1.7*	896.3 ± 53.3*
**100**	7	0.7243 ± 0.023	86.4 ± 0.7	70.4 ± 10.5
	14	12.553 ± 0.358*	71.5 ± 1.0*	1167.3 ± 89.6*
**150**	7	0.805 ± 0.056	89.1 ± 1.2	75.3 ± 5.8
	14	12.160 ± 0.765*	78.9 ± 2.4*	1120.5 ± 90.6*
**200**	7	0.583 ± 0.032	91.4 ± 1.1	55.9 ± 2.7
	14	10.902 ± 0.141*	81.3 ± 0.5*	917.0 ± 11.6*
**400**	7	0.506 ± 0.05	93.0 ± 0.9	49.6 ± 0.4
	14	7.201 ± 0.042*#	87.7 ± 0.7*	643.8 ± 5.6*#

*P < 0.05 compared to day 7

**P < 0.05 comparing UM171 at 400 nM to all other UM171 doses

### Effect of 14-day CD34 + cell expansion on stem cell phenotype

**Fig. 1:**
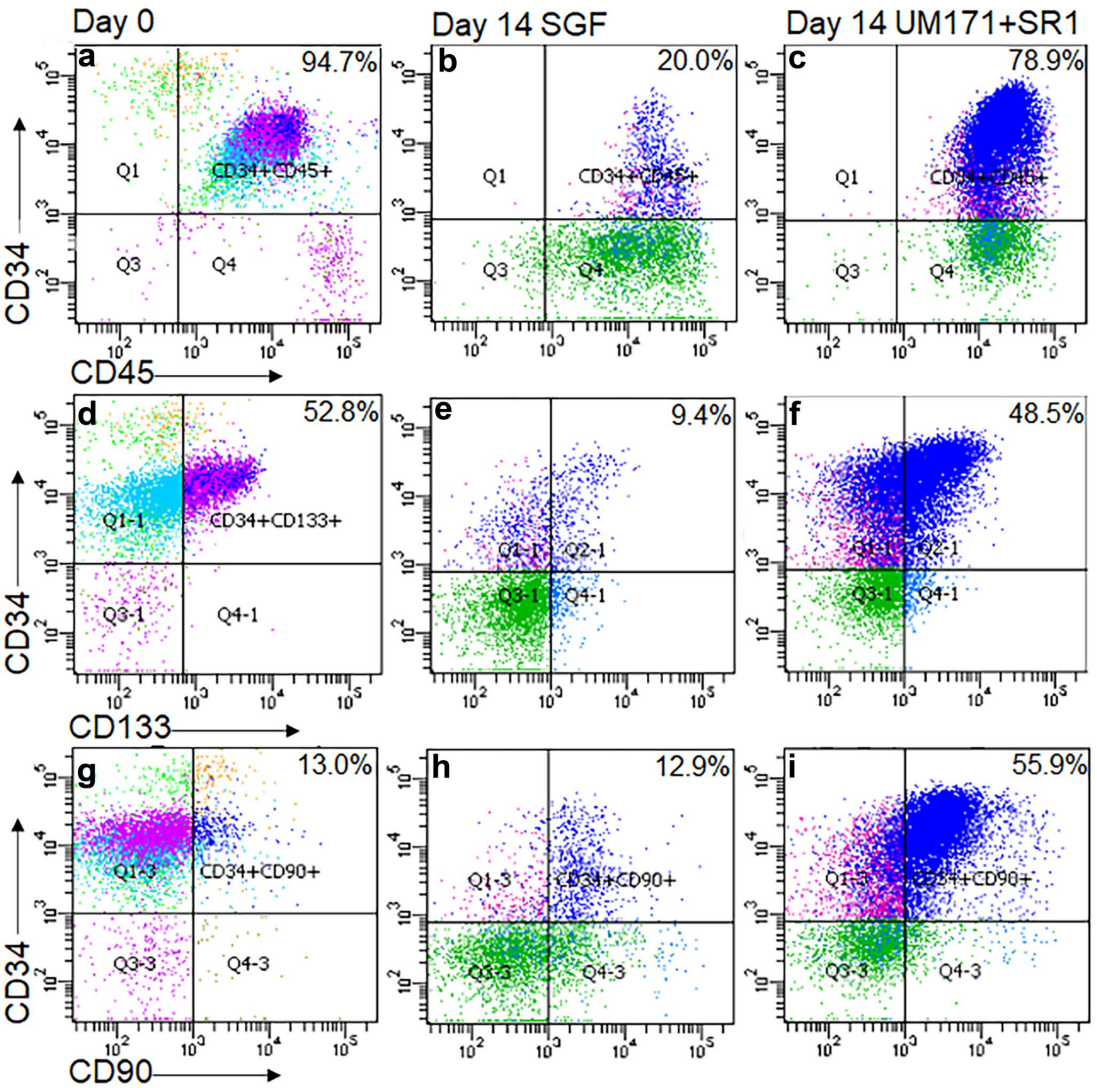
Stem cell phenotype analysis. Flow cytometry analysis of CD34 + cells prior to expansion (day 0) and after 14 days of expansion in either SGF containing media or UM171 + SR1 containing media for co-expression of CD34/CD45 + (**a-c**), CD34/CD133 + (**d-f**) and CD34/CD90 + (**g-i**) populations

Phenotypical characterisation of the 14-day expanded CD34 + cells cultured in either SGF or with 150 nM UM171 + and 750 nM SR-1 was performed using flow cytometric analysis of CD34, CD45, CD133, CD90 and CD43 expression. Pre-expanded CD34 + were used with a minimum purity of 90% for CD34/CD45 + cells for all expansion experiments with an average of 94.7 ± 0.8% (Table 4). After 14 days of expansion in SGF media, the CD34/CD45 + cells were 20.0 ± 1.9%, whereas addition of UM171 + SR-1 resulted in 78.9 ± 2.4% CD34/CD45 + cells (Fig. 1a-c, Table 4). The CD34/CD133 + population, defined as both HSCs and endothelial progenitor cells (EPCs), showed that 52.8 ± 3.2% of the cells co-expressed these two markers before expansion and, after 14 days of expansion in SGF, this was significantly reduced to 9.4 ± 2.3% and in UM171/SR-1 containing media to 48.5 ± 2.7% (Fig. 1d-f, Table 4). The CD34/CD90 + population showed that 13.0 ± 2.5% of the cells co-expressed these two markers before expansion and, after 14 days of expansion in SGF, similar positivity of 12.9 ± 1.3% was observed and in UM171/SR-1 containing media significantly increased to 55.9 ± 2.4% (Fig. 1g-i, Table 4). Further analysis of cells expressing CD34/CD45/CD43/CD133/CD90 + showed that, at day 0 pre-expansion, the proportion of the CD34/CD45/CD43/CD133/CD90 + cells was 7.5 ± 2.3%, 6.6 ± 0.9% at day 14 in SGF media and significantly increased to 35.3 ± 3.0% in UM171 + SR-1 media (Table 4).

**Table 4:** Stem cell marker populations

Cell populations (% positivity)	Pre-expansion	Day 14 expanded in SGF	Day 14 expanded in UM171 + SR-1
CD34 + CD45 +	94.7 ± 0.8	20.0 ± 1.9*	78.9 ± 2.4*#
CD34 + CD133 +	52.8 ± 3.2	9.4 ± 2.3*	48.5 ± 2.7#
CD34 + CD90 +	13.0 ± 2.5	12.9 ± 1.3	55.9 ± 2.4*#
CD34 + CD45 + CD43 + CD90 + CD133 +	7.5 ± 2.3	6.6 ± 0.9	35.3 ± 3.0*#

*P < 0.05 compared to pre-expansion

**P < 0.05 UM171 + SR1 compared to SGF

### Effect of 14-day CD34 + cell expansion on cell cycle 

**Fig. 2:**
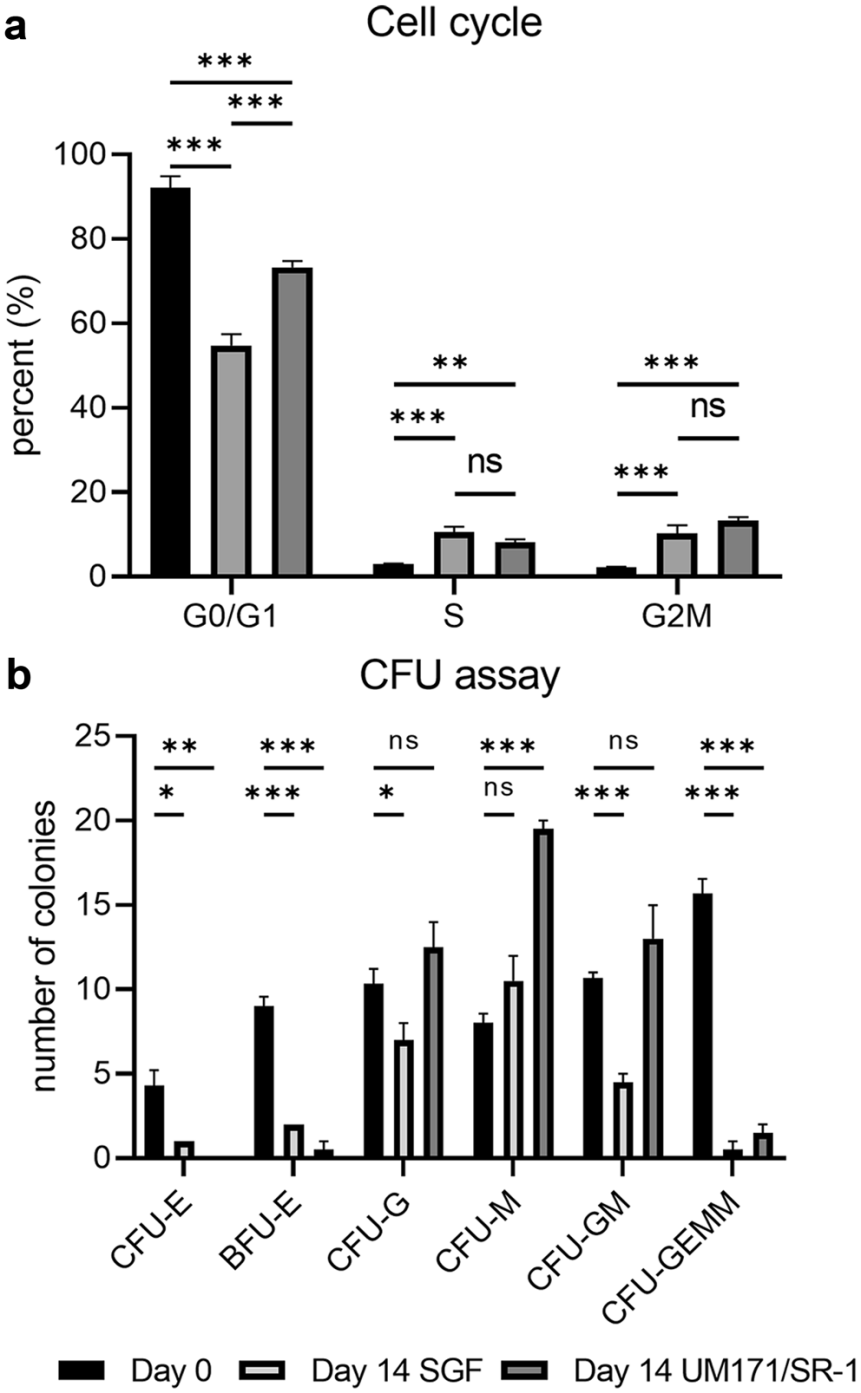
Cell cycle and colony-forming (CFU) unit assay. Flow cytometry-based cell cycle analysis of day 0 pre-expansion CD34 + cells, Day 14 expanded CD34 + cells in SGF media, Day 14 expanded CD34 + cells in UM171 + SR-1 media (**a**). CFU assay for colony counting for CFU-E, BFU-E, CFU-G, CFU-GM, CFU-GEMM (**b**). *P < 0.05, **P < 0.01, ***P < 0.001, N = 3 independent experiments

At 14 days, expansion in media containing SGF resulted in 54.8% G0/G1, 10.5% S and 10.3% G2M cells (Fig. 2a). At 14 days, with the addition of 150 nM UM171 and 750 nM SR-1, 73.3% of cells were in the G0/G1 phase, 8.1% S and 13.3% G2M. When compared to pre-expansion CD34 + cells, there were significantly less cells in G0/G1 phase in both day-14 cells expanded in SGF media (p < 0.001) and UM171 + SR-1 media (p < 0.001), but significantly more cells in G0/G1 in day-14 cells expanded in UM171 + SR-1 (p < 0.001) compared to those cultured in SGF media. For S phase, there were significantly more cells when expanded for 14 days in both SGF media (p < 0.001) and UM171 + SR-1 media (p = 0.003) compared to pre-expansion and no difference between expanded cells in SGF media compared to UM171 + SR-1 containing media (p = 0.197). For G2M phase, there were significantly more cells when expanded for 14 days in both SGF media (p < 0.001) and UM171 + SR-1 media (p < 0.001) compared to CD34 + cells pre-expansion, but no significant difference in cells expanded for 14 days in SGF or UM171 + SR-1 (p = 0.093).

### Effect of 14-day CD34 + cell expansion on colony-forming unit (CFU) assay

To assess clonal capacity of the CD34 + cells after 14-day expansion, the colony-forming unit (CFU) assay was used (Fig. 2b). For CFU-E, there was a significantly reduced colony count when compared to day 0 pre-expanded CD34 + cells; an average of 4.3 colonies at Day 0, 1.0 colony at day 14 in SGF media (p = 0.018) and no colonies observed at day 14 in media containing UM171 + SR-1 (p = 0.002). For BFU-E, the highest colony count was observed for pre-expansion, at 8.7 colonies, and a significant decline in colonies was observed for day 14 cells expanded in SGF media (1.7 colonies; p < 0.001) and day 14 cells expanded in UM171 + SR-1 media (1.4 colonies; p < 0.001). For CFU-G colonies, day 0 pre-expansion contained 10.3 colonies where day 14 SGF expanded CD34 + cells were significantly down-regulated at 7.3 colonies (p = 0.018); however, the day 14 UM171 + SR-1 expanded CD34 + cells showed no significant difference with 12.5 colonies (p = 0.143). There was no significant difference in the number of CFU-G colonies across all groups. For CFU-M, there was no difference between pre-expanded CD34 + cells and after expansion in SGF media (p = 0.083); however, expansion with UM171 + SR-1 significantly increased the number of CFU-M colonies compared to both pre-expansion and SGF expanded cells (p < 0.001). CFU-GM pre-expanded CD34 + cells resulted in 10.7 colonies and a significant decline in colonies were observed for day 14 cells expanded in SGF media (4.5 colonies) (p < 0.001); however, there was no significant difference between pre- and post-expanded CD34 + cells in UM171 + SR-1 media (12.6 colonies) (p = 0.109). For CFU-GEMM, the highest colony count was observed for pre-expansion at 15.7 colonies and a significant decline in colonies was observed for day 14 cells expanded in SGF media (0.6 colonies; p < 0.001) and UM171 + SR-1 media (1.5 colonies; p < 0.001).

### Effect of CD34 + cell expansion on neurotrophic and angiogenic factor gene expression

**Fig. 3:**
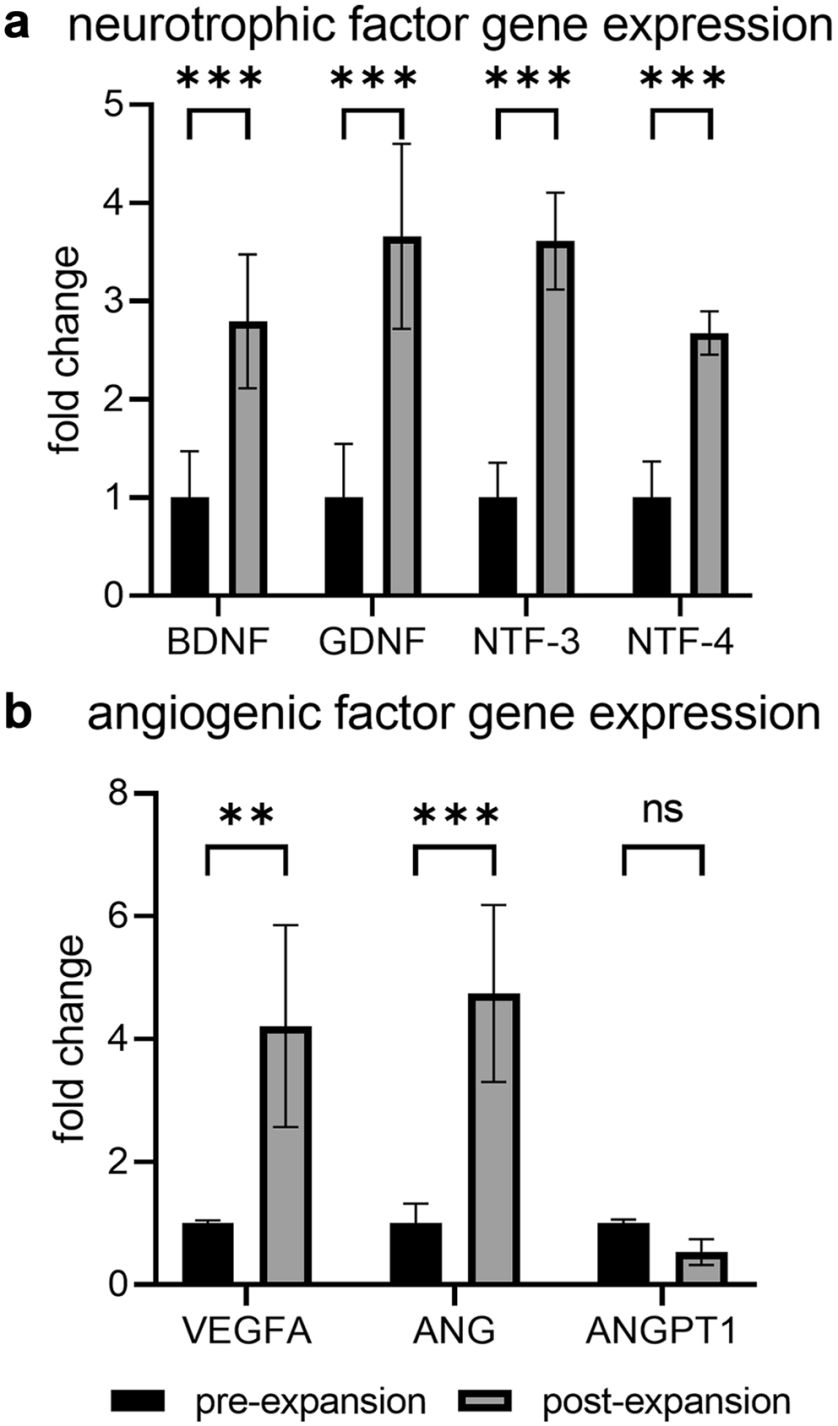
Neurotrophic and angiogenic factor gene expression. Gene expression from pre- and post-expanded CD34 + cells for neurotrophic factor gene expression for GDNF, BDNF, NTF-3 and NTF-4 (**a**). Angiogenic factor gene expression for VEGFA, ANG and ANGPT1 (**b**). Gene expression shown relative to GAPDH. **P < 0.01, ***P < 0.001 or ns—not significant, N = 3 independent experiments, performed in triplicate

Previous reports have shown that CD34 + cells express neurotrophic and angiogenic factor genes (Paczkowska et al. 2016). RT-qPCR was used to determine if a 14-day expansion using UM171 and SR-1 affected neurotrophic and angiogenic factor gene expression. CD34 + expansion with UM171 and SR-1 showed a significant upregulation of GDNF (p < 0.001), BDNF (p < 0.001), NTF-3 (p < 0.001) and NTF-4 (p < 0.001) (Fig. 3a), compared to pre-expanded CD34 + cells. For angiogenic factor gene expression, expanded CD34 + cells showed a significant up-regulation of ANG (p < 0.001) and VEGFA (p < 0.01) compared to pre-expanded cells, but no significant difference was observed for ANGPT1 (Fig. 3b).

### Expanded CD34 + cells promote tube formation in vitro

**Fig. 4:**
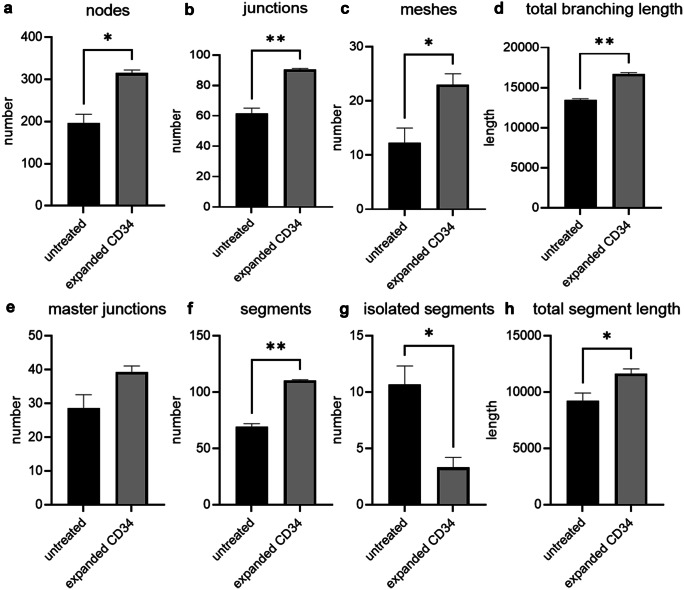
Angiogenesis tube formation assay. 14-day UM171 + SR-1 expanded CD34 + cells effect on HUVEC tube formation. Nodes (**a**), junctions (**b**), meshes (**c**), total branching length (**d**), master junctions (**e**), segments (**f**), isolated segments (**g**) and total segment length (**h**). NT non-CD34 + cell treated. *P < 0.05 and **P < 0.01, N = 3 independent experiments

To assess if 14-day expansion of CD34 + cells in the presence of UM171 + SR-1 retained their angiogenic potential, a tube formation assay was used (Fig. 4). Expanded CD34 + cells showed a significant increase in the numbers of nodes (p = 0.017, Fig. 4a), junctions (p = 0.0076, Fig. 4b), meshes (p = 0.032, Fig. 4c) and segments (p = 0.0038, Fig. 4f) but not master junctions (p = 0.068, Fig. 4e), compared to the untreated control. The addition of expanded CD34 + cells significantly decreased the number of HUVEC isolated segments (p = 0.017, Fig. 4g), which are too small to form branches/segments. However, the expanded CD34 + cells significantly increased the total branching length (p = 0.0067) and the total segment length (p = 0.04) when compared to untreated control (Fig. 4d and h).

### Expanded CD34 + cells promote glial cell proliferation

**Fig. 5:**
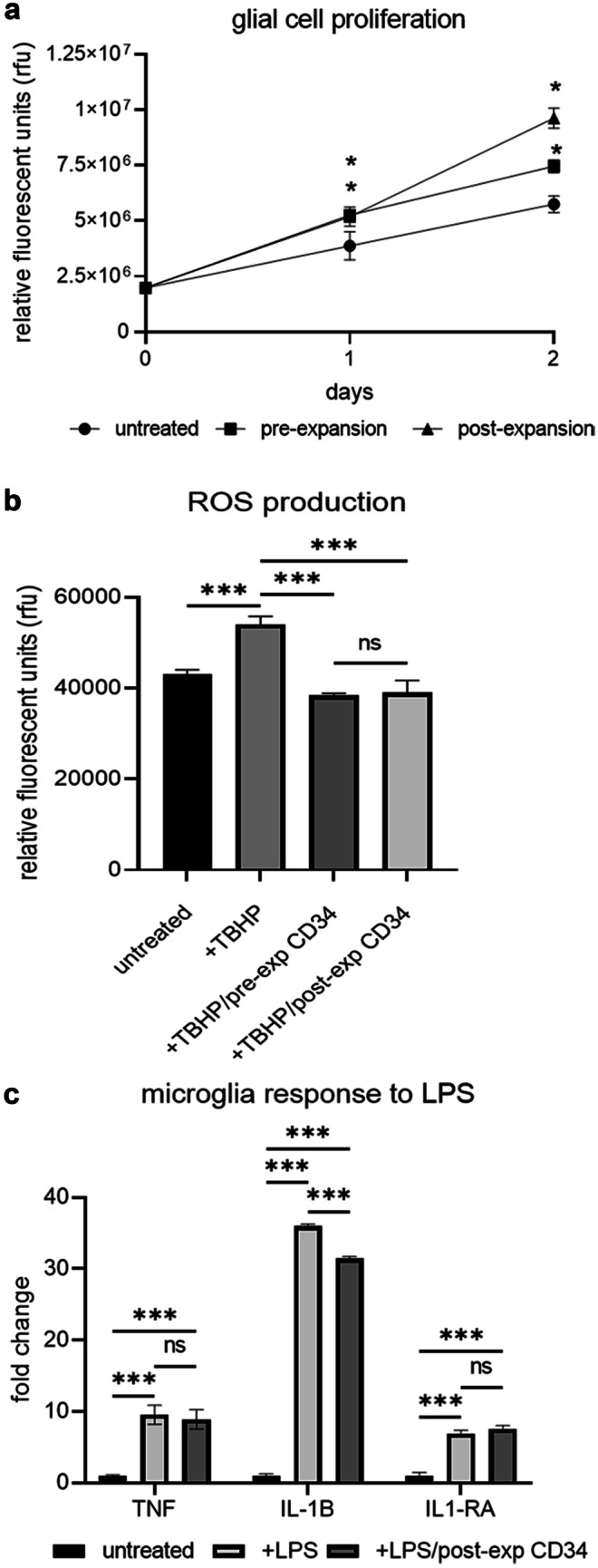
Glial cell proliferation and microglia anti-inflammatory assay. Effect of CD34 + cells on glial cell proliferation (**a**). TBHP induction of oxidative stress comparing untreated cells (NT) and the addition of TBHP with either pre- or post-expanded CD34 + cells (**b**). Effect of CD34 + cells to augment cytokine gene expression on microglia challenged with LPS (**c**). *P < 0.05, **P < 0.01, ***P < 0.001. N = 3 independent experiments

Mixed glial cells were isolated from the cortex of post-natal day 3 rat pups and used for proliferation assays. Pre- or post-expansion CD34 + cells were placed in 0.4 µM transwells above the glial cell monolayer and cultured over two days. After 1 day, both the pre- and post-expanded CD34 + cells significantly promoted proliferation of the glial cells (p = 0.003 and p = 0.004, respectively) and after 2 days of culture this was further increased with pre-expanded CD34 + cells (p < 0.001) and post-expanded CD34 + (p < 0.001) further increasing cell proliferation. The greatest increase in glial cell proliferation was observed in the post-expanded CD34 + group when compared to pre-expanded CD34 + cells (p < 0.001, Fig. 5a).

### Expanded CD34 + cells can attenuate ROS production during oxidative stress

Oxidative stress has a negative impact on cells in the brain, and reactive oxygen species (ROS) are generated in response to hypoxia ischemia. In this study, we tested the ability of CD34 + cells to provide anti-oxidant support to glial cells that had been exposed to 5 µM tert-butyl hydroperoxide (TBHP) which causes oxidative stress (Roy and Sil 2012). There was a significant upregulation of ROS production in glial cells when exposed to TBHP (p < 0.001) compared to untreated cells (Fig. 5b). Treatment of the glial cells with TBHP along with non-expanded CD34 + cells or 14-day expanded CD34 + cells resulted in a significant down-regulation of ROS generation from the glial cells (p < 0.001). There was no significant difference between non-expanded CD34 or expanded CD34 cells in their ability to attenuate ROS (p = 0.95).

### Effect of expanded CD34 + cells on cytokine response in LPS stimulated microglia

Microglia were isolated by selection of CD11b + cells from the cortex of post-natal day 3 rat pups. When microglia were challenged with LPS, there was a significant up-regulation of pro-inflammatory genes; TNF (p < 0.001) and IL-1β (p < 0.001) and of the anti-inflammatory IL-1RA (p < 0.001) compared to the untreated control (Fig. 5c). When co-cultured with expanded CD34 + cells, there was no significant difference in microglial production of TNF and IL-1RA gene expression compared to microglia + LPS. However, co-culture of expanded CD34 + cells was able to significantly downregulate expression of IL-1β (p < 0.001) compared to microglia + LPS.

## Discussion

Umbilical cord blood is a rich source of HSCs; however, the number of HSCs obtained from single UCB units may not be sufficient for regenerative medicine applications, particularly if cells are to be given to an adult or multiple doses are required. Cell expansion addresses this limitation for the use of HSC cell-based therapies by generating sufficient cell numbers, but up until now it has not been determined if ex vivo expansion of HSCs impacts the paracrine secretions and function of these cells. Accordingly, in the current study we addressed this knowledge gap by carrying out cell expansion of UCB CD34 + cells using various individual and combined doses of standard growth factors (TPO, SCF, IL-6 and FLT-3), LDL and the small molecules (UM171 and SR-1) over 7 or 14 days. Our results have shown that UCB CD34 + cell expansion is achievable with greater than 1000-fold cell expansion of CD34/CD45 + cells at 14 days when both small molecules UM171 and SR-1 were added to the media. Expansion was significantly greater at 14 days compared to 7 days, within the order of 10 × greater total cell numbers. Finally, we describe here for the first time that expansion of CD34 + cells with TPO, SCF, FLT-3, IL-6, UM171 and SR-1 led to an up-regulation of the neurotrophic factors GDNF, BDNF, NTF-3 and NTF-4 as well as the angiogenic factors VEGF and ANG, but not of ANGPT1. In addition, we have shown, in vitro, that expanded CD34 + cells improve angiogenesis and glial proliferation and significantly reduce oxidative stress and microglial IL-1B expression.

Our results demonstrate that the addition of UM171 and SR-1 to SGF (TPO, IL-6, SCF and FLT-3) supported self-renewal of CD34 + -derived cells over a two-week expansion culture when compared to SGF or either UM171 or SR-1 added individually, which was most likely due to the fact that both molecules target different pathways (Boitano et al. 2010; Chagraoui et al. 2021). Previous expansion studies using TPO, FLT-3, IL-6, IL-3 and SCF (Petzer et al. 1996) resulted in poor expansion and differentiation of CD34 + cells. Methods to try to address this issue have used co-cultures containing mesenchymal stromal cells to recapitulate the HSC niche (Jing et al. 2010; Kadekar et al. 2015) or addition of factors including notch ligands (Delaney et al. 2010), FGF (de Haan et al. 2003), pleiotrophin (Himburg et al. 2010), angiopoietin-like proteins (Zhang et al. 2006), valproic acid (De Felice et al. 2005), low density lipoproteins (LDL) (Cimato et al. 2013), GSK-3β inhibitors (Ko et al. 2011) and nicotinamide (Horwitz et al. 2014). More recently, increased attention has focused on the use of small molecules to regulate stem cell fate. Of interest with respect to regulation of HSC expansion are the small molecules SR-1, an aryl hydrocarbon receptor antagonist (Boitano et al. 2010) and UM171, a pyrimidoindole derivative (Fares et al. 2014). These small molecules have shown promising results in their ability for ex vivo expansion of HSCs where the addition of SR-1 results in a 50-fold increase in HSCs (Boitano et al. 2010) and UM171 resulted in expansion of HSCs by up to 100-fold in 7 days of culture. These expanded cells also showed the ability for long-term repopulation in vivo either in combination with SR-1 or when added alone (Fares et al. 2014). The discovery of these small molecules has led to advances in the clinical use of small molecule expanded HSCs for use in haematological indications (Wagner et al. 2016; Cohen et al. 2020; Dumont-Lagacé et al. 2020).

In our CD34 + expansion culture system, we found that the optimal UM171 concentration of 150 nM maintained CD34 + HSC self-renewal without negatively impacting proliferation and their secretome. The number of HSCs that can be generated during ex vivo expansion is a function of the cell fate such as quiescence, proliferation, differentiation, and death which, in turn, can be regulated ex vivo by extrinsic factors (Domen and Weissman 1999; Bonde et al. 2004; Ross and Li 2006). The expansion protocol described in this study used 1 × 10^4^ CD34 + cells as a starting point in a 24 well format and, by day 7, the cells reach approximately 80% confluence. These cells were split at a ratio of 1:20 and further cultured for an additional 7 days. This passaging at day 7 allowed the cells to continue to rapidly proliferate and resulted in a total expansion of CD34 + CD45 + cells of 1120-fold at day 14, when SR-1 and UM171 were added to the media at a dose of 150 nM. 14 day expansion using UM171 combined with SR-1 resulted in the loss of CFU-E colony-forming potential, which is consistent with the findings that UM171 alone can inhibit erythroid and megakaryocyte differentiation (Fares et al. 2014). However, the 14-day expansion with UM171 + Sr-1 resulted in the gain of CD34 + CD45 + CD43 + CD90 + progenitors, whereas expansion without UM171 and SR-1 resulted in a lower yield of these progenitors. This population of progenitors has been suggested to be a more primitive HSC and is capable of long-term repopulating activity (Radtke et al. 2020) suggestive that the expansion set out in this study promotes expansion of a primitive phenotype of HSC.

CD34 + cells have been well characterised for their ability to promote angiogenesis by expression of angiogenic factors (Scheubel et al. 2010; Sahoo et al. 2011; Mathiyalagan et al. 2017). In in vivo animal models, treatment of hypoxic ischemic brain injury with CD34 + cells improved vascular regeneration and cerebral blood flow, as well as a reduction in apoptosis (Ohshima et al. 2016; Yu et al. 2019). The mechanisms of action of CD34 + cells for repair and regeneration is suggested to be due to paracrine action, rather than engraftment and differentiation of the cells at the site of injury. This paracrine effect stimulates proliferation of endogenous cells as well as regulation of cell survival through the secretion of growth and trophic factors such as neurotropic and angiogenic factors (Wang et al. 2013; Zhao et al. 2014; Englander et al. 2015). In our studies using UM171/SR-1 expanded UCB CD34 + cells resulted in the significant upregulation of the angiogenic factors VEGF and ANG, but not ANGPT1, suggestive that 14-day expanded CD34 + cells are not negatively impacted in their ability to promote angiogenesis. To further assess this capacity, we used a tube formation assay using HUVECs co-cultured with the CD34 + cells. We showed that the addition of CD34 + cells promoted tube formation with increased numbers of segments, junctions and meshes, as well as increased length of the segments and junctions, while there was a significant decrease in the number of isolated segments (incomplete segments). This suggests that a 14-day expansion did not negatively affect CD34 angiogenic activity in vitro.

Neurotrophic factors are a family of proteins that regulate proliferation, differentiation, survival and apoptosis in cells of the nervous system (Hefti et al. 1993; Thorne and Frey 2001). Neurotrophic factors have been directly linked to preventing neuronal cell death in the injured brain (Kubo et al. 1995; Schäbitz et al. 2007; Fantacci et al. 2013; Chen et al. 2019). Studies have shown unmanipulated bone marrow-derived CD34 + cells express higher levels of the neurotrophic factors NGF, BDNF, GDNF, NTF-3 and NTF-4, as well as their receptors Trk-A, Trk-B, P75NTR when compared to bone marrow-derived nucleated cells (Paczkowska et al. 2016). However, the impact of expansion on trophic factor expression has not previously been assessed. Here, we describe for the first time that expansion of CD34 + cells with TPO, SCF, FLT-3, IL-6, UM171 and SR-1 led to an up-regulation of the neurotrophic factors GDNF, BDNF, NTF-3 and NTF-4. This up-regulation of neurotrophic factors could be due to the specific factors used to expand the CD34 + cells which may have promoted the up-regulation of the genes we assessed or, the upregulation could be caused by the change in phenotype of the CD34 + cells as expansion does result in some maturing of CD34 + cells over time.

Our results demonstrated that expanded CD34 + cells up-regulated neurotrophic factors which can have a role in regenerative processes, and we next assessed the impact of these cells on glial cells isolated from the brains of three-day old rat pups. Growth factors that are secreted by HSCs have been shown to regulate proliferation, differentiation, and release of blood cell lineages into the circulation (Majka et al. 2001; Paczkowska et al. 2016). These growth factors have also been shown to regulate the proliferation of neural stem/progenitor cells (Cattaneo and McKay 1990; Arsenijevic et al. 2001; Younsi et al. 2020). Results from our study found that both pre- and post-expanded CD34 + cells significantly increased glial cell proliferation; however, the expanded CD34 + cells caused greater proliferation in the glial cells than the pre-expanded cells, suggestive that the higher proliferation may be correlated to the up-regulation of neurotrophic factors after expansion. A number of studies using pre-clinical animal models have investigated the neuroprotective/neuroreparative potential of UCB cells for treating hypoxic ischemic brain injury in neonates and adult. These studies report that UCB cell administration has neuroprotective/neuroregenerative benefits with improvements in motor control and coordination, as well as reduced neuronal degeneration and apoptosis, and that UCB cells protect neuronal and glial cell populations in response to preterm and term brain injury (Ohshima et al. 2016; McDonald et al. 2018; Paton et al. 2018; Yu et al. 2019). It is possible that the CD34 + cells present in UCB provide a neuroprotective and functional role and that our expanded cord blood cells could have the potential to provide a therapeutic benefit in neuroprotective/neuroreparative applications.

Cells of the myeloid lineage and resident microglia in the brain play a major role in the pathogenic processes that contribute to tissue damage in brain injury by promoting neuroinflammation and oxidative stress (Tichauer et al. 2007; Rosales-Corral et al. 2010; Pathipati et al. 2013; Bhalala et al. 2015). Here, we showed that when cultured microglia were stimulated with the inflammatory stimulator LPS, the inflammatory cytokines TNF, and IL-1β were up-regulated along with the anti-inflammatory IL-1RA. When microglia were co-cultured with expanded CD34 + cells, there was no difference in TNF or IL-1RA gene expression levels, but there was a significant down-regulation of the pro-inflammatory cytokine IL-1β, suggesting that expanded CD34 + cells are also anti-inflammatory.

Finally, we showed that UCB expanded CD34 + cells could attenuate reactive oxygen species (ROS) production in rat glial cells that were treated with TBHP, an oxidative stress inducer (Roy and Sil 2012). When comparing pre- and post-expanded CD34 + cells, there was no difference in their ability to down-regulate ROS, indicating that expansion of the cells does not negatively impact the anti-oxidant capacity of CD34 + cells. Oxidative stress caused by pathological changes can result in the over production and impaired clearance of ROS, a key mechanism contributing to tissue damage and functional deficits in hypoxic-ischemic brain injury (Ten and Starkov 2012; Vasiljević et al. 2012; Zhao et al. 2016). It is possible that the effect of CD34 + cells on oxidative stress in glial cells could also be due to providing anti-oxidant support to the glial cells, which remains to be confirmed in future experiments.

In summary, UCB-derived CD34 + HSCs, cultured using a 14-day expansion protocol, showed a robust expansion capacity that supports the therapeutic use of such cells. The 14-day expanded UCB-CD34 cells retain functionality when tested in in vitro assays where they promoted cell proliferation, attenuated ROS production caused from oxidative stress and promoted angiogenesis. Future studies will confirm the therapeutic potential of these cells in vivo and the scale up method for production of GMP compliant cells for therapeutic regenerative medicine clinical trials.
